# The Accumulation of Organic Carbon in Mineral Soils by Afforestation of Abandoned Farmland

**DOI:** 10.1371/journal.pone.0032054

**Published:** 2012-03-06

**Authors:** Xiaorong Wei, Liping Qiu, Mingan Shao, Xingchang Zhang, William J. Gale

**Affiliations:** 1 State Key Laboratory of Soil Erosion and Dryland Farming in the Loess Plateau, Northwest A & F University, Yangling, China; 2 College of Resources and Environment, Northwest A & F University, Yangling, China; DOE Pacific Northwest National Laboratory, United States of America

## Abstract

The afforestation of abandoned farmland significantly influences soil organic carbon (OC). However, the dynamics between OC inputs after afforestation and the original OC are not well understood. To learn more about soil OC dynamics after afforestation of farmland, we measured the soil OC content in paired forest and farmland plots in Shaanxi Province, China. The forest plots had been established on farmland 18, 24, 48, 100, and 200 yr previously. The natural ^13^C abundance of soil organic matter was also analyzed to distinguish between crop- and forest-derived C in the afforested soils. We observed a nonlinear accumulation of total OC in the 0–80 cm depth of the mineral soil across time. Total soil OC accumulated more rapidly under forest stands aged 18 to 48 yr than under forest stands aged 100 or 200 yrs. The rate of OC accumulation was also greater in the 0–10 cm depth than in the 10–80 cm depth. Forest-derived OC in afforested soils also accumulated nonlinearly across time, with the greatest increase in the 0–20 cm depth. Forest-derived OC in afforest soils accounted for 52–86% of the total OC in the 0–10 cm depth, 36–61% of the total OC in the 10–20 cm depth, and 11–50% of the total OC in the 20–80 cm depth. Crop-derived OC concentrations in the 0–20 cm depth decreased slightly after afforestation, but there was no change in crop-derived OC concentrations in the 20–80 cm depth. The results of our study support the claim that afforestation of farmland can sequester atmospheric CO_2_ by increasing soil OC stocks. Changes in the OC stocks of mineral soils after afforestation appear to be influenced mainly by the input of forest-derived C rather than by the loss of original OC.

## Introduction

Changes in land use have important effects on ecological processes and global climate change [1], [2], [3]. During the past two decades, many studies have focused on the effects of land use change on the carbon content of ecosystems, particularly in soils. These effects are not well understood due to large differences in climatic conditions, soil properties, and the type of land use change [2], [4]–[12].

Forest ecosystems perform important ecological functions and are essential for maintaining life on local and global scales [13]. The afforestation of abandoned farmland has occurred in many parts of the world due to an increasing demand for timber and wood products, the high economic returns from forestry, the desire to protect soil and water resources, and the potential of forests to sequester carbon to counter climatic change [13], [14]–[16]. For example, the afforestation of large areas throughout New Zealand have created a major C sink, offsetting about 59% of the country's greenhouse gas emissions (CO_2_-equivalent) from energy and industrial uses and 29% of country's total gas emissions in 2006 [16]. Similarly, more than 20.7 million ha of abandoned farmland have been afforested in China through the “Grain-for-Green Project”, which aims to reduce soil loss and land degradation in the western part of the country [17].

The large potential for CO_2_ sequestration in soil after the afforestation of farmland has been widely acknowledged [5], [11], [18]. Generally, soil organic carbon (OC) declines in the first years after afforestation and then gradually increases [11], [19], [20]. However, the stand age in most studies has been less than 60 yrs. This is insufficient for a thorough understanding of OC dynamics after the afforestation of farmland.

Assessing the response of soil OC to land use change requires an understanding of the dynamics of OC derived from both the previous and the current land use forms. Many researchers have reported on C dynamics after land use change [21]–[25]; however, knowledge about the dynamics of crop-derived OC after the afforestation of farmland is lacking. Such information is essential for accurately evaluating OC changes in soils and precisely predicting C sequestration by afforestation. Additionally, the dynamics of soil OC in mineral soils after the afforestation of farmland is unclear and needs further attention. In this experiment, we combined a chronosequence approach with differences in the ^13^C natural abundance of C_3_ (forest) and C_4_ (crop) plants to study the dynamics of original crop-derived OC and newly input forest-derived OC in afforested farmland.

## Materials and Methods

### Ethics Statement

The permit for soil sampling was obtained from the Huanglongshan Forest Bureau, which is responsible for the protection and management of the Huanglongshan Forest Area.

### Study site

**Figure 1:**
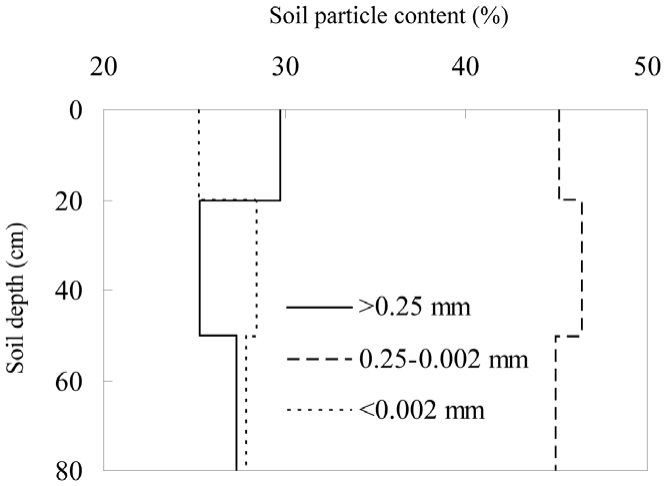
Soil particle distribution in the study site.

We performed this study in the Huanglongshan Forest Area, which is located in central Shaanxi Province, China (109°38′49″–110°12′47″E, 35°28′49″–36°02′01″N, 1100 to 1300 m above sea level). The area has a continental, monsoon climate with an average annual temperature of 8.6°C. Mean monthly temperatures range from −22.5°C in January to 36.7°C in July. The average annual precipitation is 612 mm, and the average frost-free period is 175 days. The loess-derived soil in the study area is classified by Chinese scientists as a cinnamon soil, which belongs to the Cambisol soil group according to the FAO system. The soil texture is silt loam. The particle size distribution is shown in Fig. 1. The soil profile is free of stones to a depth of 50 m.

Agricultural activity in the Huanglongshan Forest Area has a history of more than 2000 yrs. The main crops are maize (*Zea mays L*) and millet (*Panicum miliaceum L*) (both C_4_ plants). Some of the farmland was converted to forest in the past, either through natural succession or through ecological restoration programs. The main tree species in these forests is Chinese pine (*Pinus tabuliformis*).

### Field investigation and soil sampling

We used the space-for-time substitution method to estimate the effects of afforestation on soil OC dynamics. The method is an important and often necessary tool for studying the temporal dynamics of plant communities and soil development across multiple timescales [26]. The study consisted of fifteen paired forest and farmland sites. The forests had been converted from farmland 18, 24, 48, 100, and 200 yrs previously. There were three replications of each age sequence, making a total of 30 sites (5 ages×2 land use types×3 replicates). The replications were 2 to 5 km apart. The forests with stand ages of 100 and 200 yrs were established by natural succession and the forest age was determined by Yang and Hou (2005) [27]. The forests with stand ages of 18, 24, and 48 yrs were man-made and identified with the help of the Huanglongshan Forest Bureau. The paired forest and farmland sites adjoined each other; therefore, we attributed differences in soil OC concentration and OC stock between paired farmland and forest to afforestation.

In this study, we assumed that there has been no significant change in soil OC concentrations of farmland across time (i.e., the soil OC of current farmland is equal to that when the farmland was abandoned 48, 100 or 200 yrs ago). We made this assumption even though there have been changes in fertilization and tillage practices over time (e.g., chemical fertilizer replaced manure as the primary source of plant nutrients about 50 yr ago). A preliminary study indicated that there had been no significant change in the soil OC of farmland during the past 26 yrs (data not shown); however, there is no direct way to quantify soil OC concentrations 100 or 200 yrs ago.

Three sampling plots were established within each treatment in August 2009. The sampling plots were 20×20 m in afforested areas and 5×5 m farmland areas. The sampling area of the afforested plots was larger than that of the farmland plots because soil variability is greater in afforested areas than in cropped areas. Each sampling plot was at least 40 m from the boundary to reduce the possibility of tree litter being added to farmland plots. The tree density and vegetation biomass was determined for each forest plot. The dominant plants on the forest floor were bunge needlegrass (*Stipa bungeana Trin.*) and Dahurian bush clover (*Lespedeza daurica (Laxm.) Schindl.*). Five trees were randomly selected to determine height, diameter at breast height (DBH, measured at 1.35 m), and crown density. The main characteristics of the vegetation in the afforested plots are presented in Table 1.

**Table 1:** Characteristics of the forest stands at Huanglongshan.

Stand age (yrs)	Tree density (trees/ha)	DBH^a^ (cm)	Height (m)	Crown density^b^	Vegetation biomass (tons ha^−1^)
18	4500	8	6	>90%	0.61
24	4000	10	8	>90%	0.69
48	3000	21	15	>95%	0.74
100	2400	32	18	>98%	0.41
200	2400	41	20	>98%	0.46

a DBH, diameter at breast height.

b Crown density was estimated visually based on the amount of light passing through the canopy, with larger values indicating a more closed canopy.

In each sampling plot, a pit 1.0 m long×0.7 m wide×1.0 m deep was dug for measuring soil bulk density. The soil was sampled using a stainless-steel cutting ring (5.0 cm diam.×5.0 cm high) at depths of 0–10, 10–20, 20–40, 40–60, and 60–80 cm. Three representative soil profiles were randomly selected in each plot, and the organic layers were removed. Samples of the mineral soil were collected with a tube auger (5.0 cm diam.) at the same depth increments as the bulk density samples. The soil samples were taken in 10 cm increments to avoid compaction. Visible pieces of undecomposed organic matter were removed. The moist soil samples were brought to the laboratory, air-dried, and ground for laboratory analysis. Forest litter was collected in each plot, brought to the laboratory, oven dried at 65°C, and ground for δ^13^C analysis.

### Laboratory and data analyses

Soil OC concentrations were analyzed with a VARIO EL III CHON analyzer (Elementar, Germany) at the Testing and Analysis Center, Northwest University, China. The soils were treated with HCl to remove the carbonates before analysis for soil OC. The OC stock was calculated as follows:
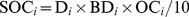
(1)where SOC*_i_*, D*_i_*, BD*_i_*, and OC*_i_* represent the OC stock (Mg ha^−1^), thickness (cm), bulk density (g cm^−3^), and OC concentration (g kg^−1^), respectively, of the *i*th layer of soil.

The natural abundance of ^13^C in the soil organic matter and forest litter was analyzed with a MAT253 Stable Isotope Ratio Mass Spectrometer (Thermo Fisher Scientific, American) at the State Key Laboratory of Hydrology-Water Resources and Hydraulic Engineering, Hohai University, China. The δ^13^C values of the soil OC and litter were calculated as follows:
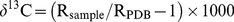
(2)where R is the ratio of ^13^C to ^12^C, and PDB is the belemnite carbonate standard of the Peedee Formation, South Carolina.

For afforested soils, the proportion (*f*) of OC derived from forest litter (C_3_-C) at each soil depth was calculated according to the following equation:
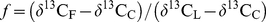
(3)where δ^13^C_F_ is the δ^13^C value of soil organic matter in afforested soil, δ^13^C_C_ is the δ^13^C value of soil organic matter in farmland soil, and δ^13^C_L_ is the δ^13^C value in forest litter (we used a mean value of −27.2‰ for the calculation). Finally, the forest-derived C (C_3_-C) and original crop-derived C (C_4_-C) in afforested soils were calculated as follows:
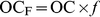
(4)

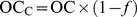
(5)

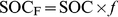
(6)

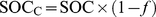
(7)where OC_F_ and OC_C_ are the concentrations of forest-derived C and crop-derived C, respectively; SOC_F_ and SOC_C_ are the stocks of forest-derived C and crop-derived C, respectively; and OC and SOC are the total OC concentrations and OC stocks in afforested soil, respectively.

To assess the dynamics of original farmland C after afforestation, we calculated the mean residence time (MRT) and half-life (T_1/2_) of the original farmland C in afforested soils. The mean residence time was calculated as [28]:
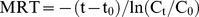
(8)where t is the time of sampling (yr), t_0_ is the time of afforestation (yrs), ln is the natural logarithm, C_t_ is crop-derived OC in the afforested soil at the time of sampling (Mg ha^−1^), and C_0_ is the crop-derived OC at the time of afforestation (Mg ha^−1^). We used the soil OC of the paired adjacent farmland as C_0_ and regressed t-t_0_ versus ln(C_t_/C_0_). The slope of the resulting line was equal to the MRT.

The T_1/2_ was calculated as:
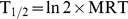
(9)The analyses of variance, correlation, and regression were conducted using SAS software (Version 8.0) [29]. Three-way analysis of variance was used to test the effects of afforestation, stand age, and soil depth on OC concentration and stock. Two-way analysis of variance was used to test the effects of stand age and soil depth on crop- and forest-derived OC concentrations and OC stocks. Correlation analysis was used to assess the relationships between forest-derived OC and the net increase in the OC of the afforested soil, and between the original OC of the afforested soil (C_4_-C) and the OC of current farmland. Regression analysis was used to establish the relationships between stand age and different variables, including OC accumulation rate and the proportion and accumulation rates of forest-derived OC in afforested soils at each soil depth.

## Results

### Increase in total OC

**Figure 2:**
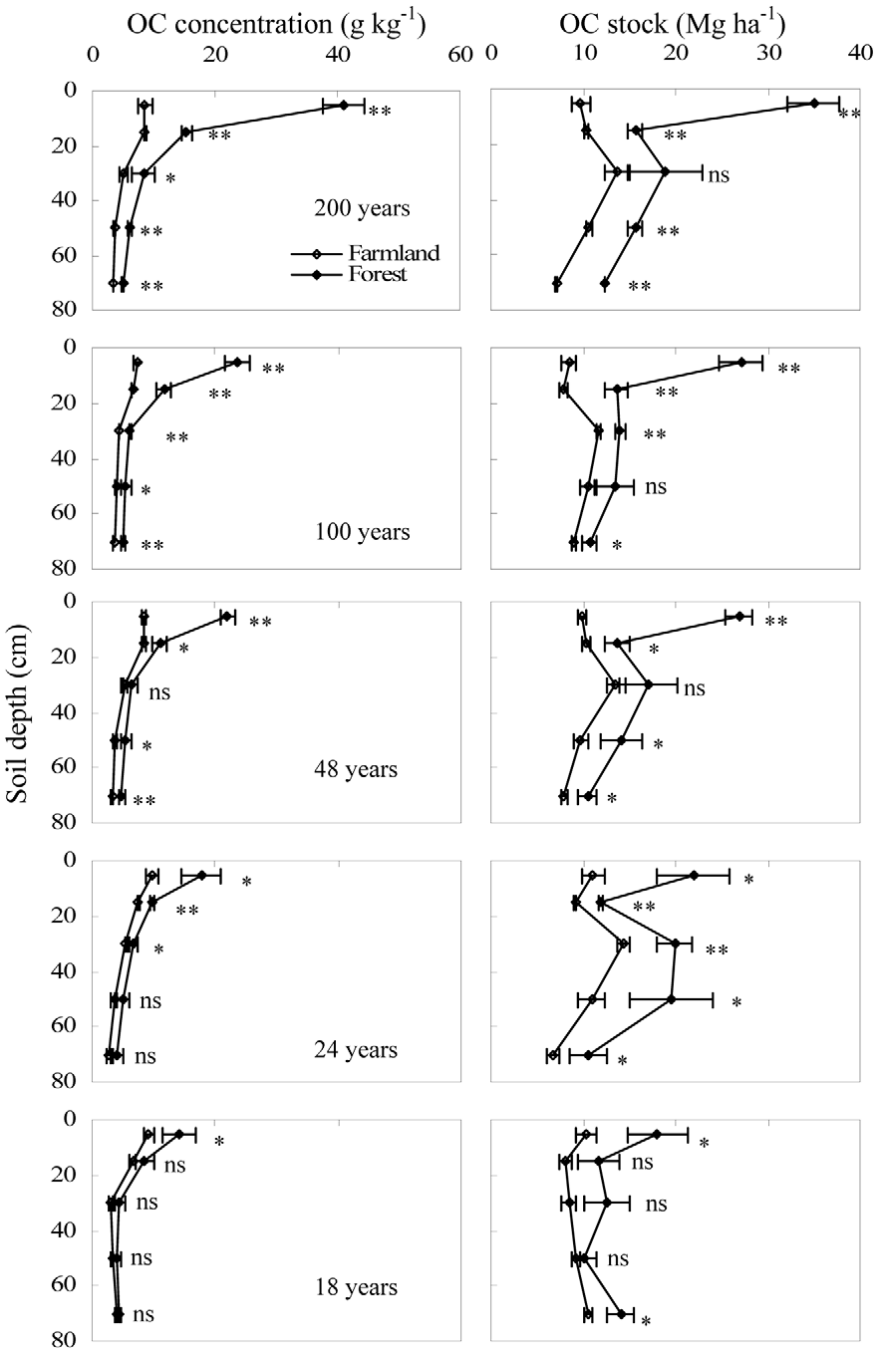
Effects of afforestation of abandoned farmland on soil OC concentration and stock at different stand ages (*: the difference between forest and farmland was significant at P<0.05; **: the difference was significant at P<0.01; ns: P>0.05; the error bars are standard deviation of the means).

The afforestation of abandoned farmland significantly increased total OC concentrations and stocks in mineral soils. The amount of increase varied with forest age and soil depth (Table 2). The increase in total OC after afforestation mainly occurred in the 0–10 cm depth. For the 0–10 cm depth, the increase in OC concentrations ranged from 5.0 to 32 g kg^−1^ and the increase in OC stocks ranged from 8.0 to 25 Mg ha^−1^. For the 10–80 cm depth, the increase in OC concentrations ranged from 0.3 to 6.8 g kg^−1^ and the increase in OC stocks ranged from 1.2 to 5.8 Mg ha^−1^ (Fig. 2).

**Table 2:** The ANOVA results of soil OC concentration and OC stock as affected by afforestation, stand age, and soil depth.

		OC concentration		OC stock	
	DF^a^	F	P	F	P
Afforestation	1	742	<0.0001	541	<0.0001
Soil depth	4	709	<0.0001	101	<0.0001
Afforestation×Soil depth	4	237	<0.0001	75	<0.0001
Stand age	4	69	<0.0001	18	<0.0001
Afforestation×Stand age	4	58	<0.0001	9	<0.0001
Soil depth×Stand age	16	23	<0.0001	8	<0.0001
Afforestation×Soil depth×Stand age	16	27	<0.0001	6	<0.0001

a DF, degrees of freedom.

**Figure 3:**
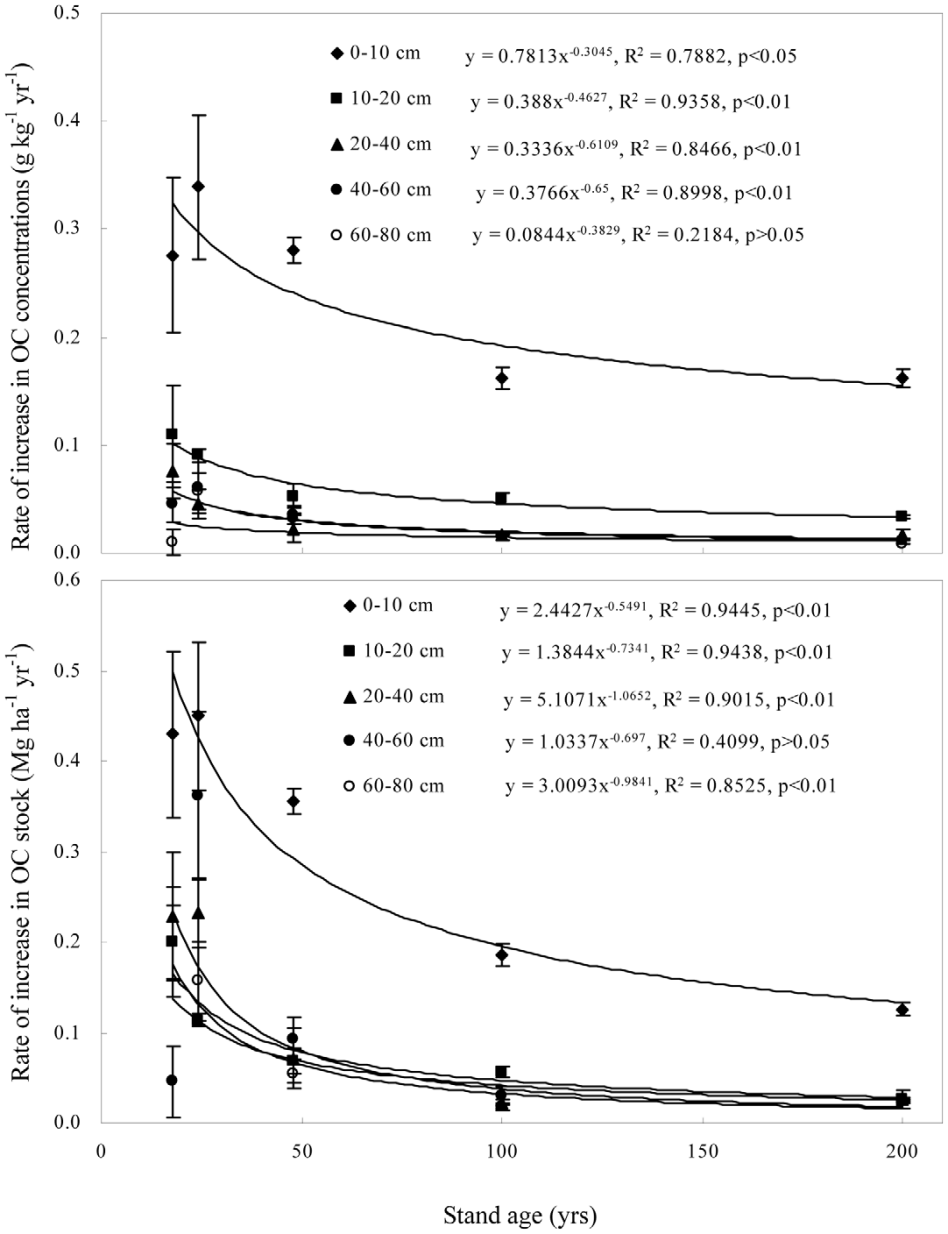
Relationships between stand age and the rate of increase in total OC concentrations and stocks in mineral soils (the error bars are standard deviation of the means).

Although total soil OC in the 0–80 cm depth increased with stand age, accumulation rates were generally highest in recently afforested soils (Fig. 3). Specifically, soil OC accumulation rates in stands less than 48 yr old ranged from 0.35 to 0.45 Mg ha^−1^ yr^−1^ for the 0–10 cm depth and from 0.06 to 0.23 Mg ha^−1^ yr^−1^ for the 10–80 cm depth. In comparison, accumulation rates in the 100 and 200 yr stands ranged from 0.13 to 0.18 Mg ha^−1^ yr^−1^ for the 0–10 cm depth and from 0.02 to 0.06 Mg ha^−1^ yr^−1^ for the 10–80 cm depth. The change in the accumulation rate with stand age was described by a power function (Fig. 3), indicating a nonlinear increase in soil OC after afforestation of farmland. The observation that accumulation rates in the 0–10 cm depth were significantly greater than those in the 10–80 cm depth across all stand ages suggests that OC inputs in the afforested soil were primarily derived from forest litter rather than tree roots.

### Increase in forest-derived OC

**Figure 4:**
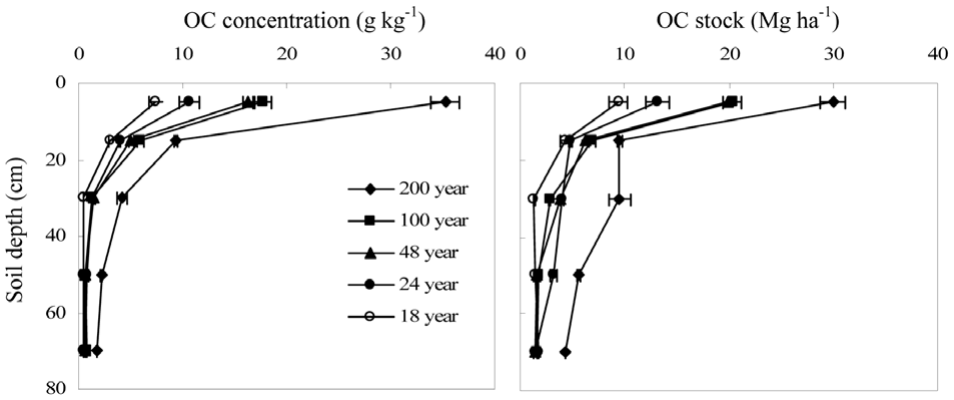
Vertical distribution of forest-derived OC (C_3_-C) concentrations and OC stocks in afforested farmland (the error bars are standard deviation of the means).

**Figure 5:**
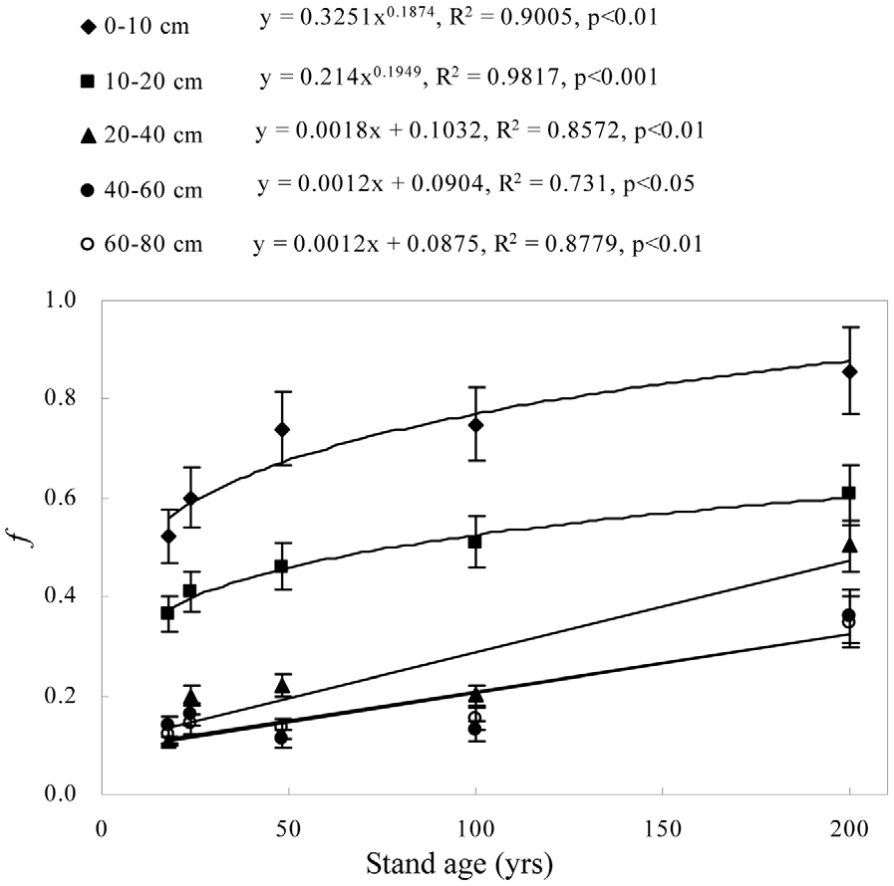
Change in the proportion (*f*) of forest-derived organic carbon to total organic carbon with different stand ages, by soil depth (the error bars are standard deviation of the means).

Forest-derived OC in afforested soils increased with stand age, with most of the increase occurring in the 0–20 cm depth (Fig. 4). Forest-derived OC comprised 52–86% of the OC in 0–10 cm depth, 36–61% of the OC in the 10–20 cm depth, and 11–50% of the OC in the 20–80 cm depth. In the 0–20 cm depth, the proportion of forest-derived C to total OC increased nonlinearly as stand age increased (Fig. 5).

**Figure 6:**
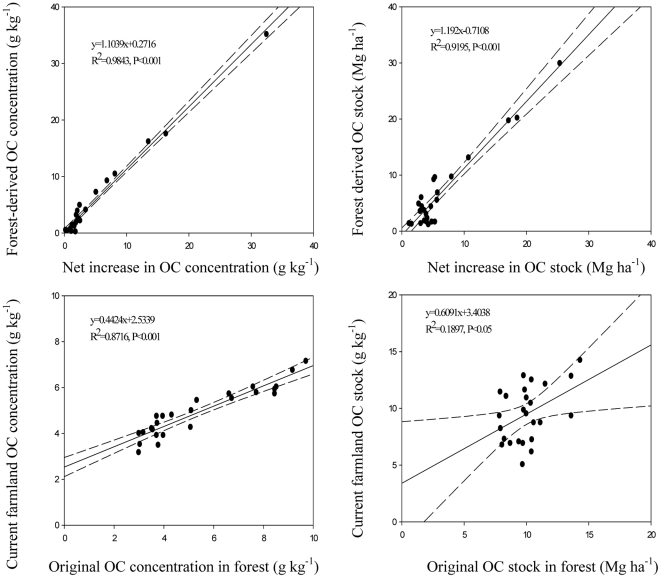
Relationships between forest-derived OC (C_3_-C) and the net increase in OC in afforested soil, and between original OC in afforested soil (C_4_-C) and the OC in current farmland (the inner bound represents the 95% confidence interval).

We assumed that the OC concentrations and OC stocks in current farmland are equal to those when the forests were first established. If this is true, then the difference in OC between forest and farmland (i.e. net increase in OC due to afforestation) should be equal to the amount of forest-derived OC determined by ^13^C analysis. We observed a highly significant linear relationship between forest-derived OC and the net increase of OC in afforested soil (Fig. 6). The slopes of the relationship were 1.1 for soil OC concentrations and 1.2 for soil OC stocks. This supports our supposition that the OC of the current farmland is equal to the OC of the farmland at the time of afforestation. The forest-derived OC concentration was on average slightly higher than the net increase in OC concentration in the 0–20 cm depth but about the same as the net increase in the OC concentration in the 20–80 cm depth. In comparison, the crop-derived OC concentrations of afforested soil were slightly less than that of current farmland at the 0–20 cm depth but about the same as that of current farmland at the 20–80 depth. These results suggest a slight decline in the crop-derived OC concentration of the 0–20 cm depth but no change in the crop-derived OC concentration of the 20–80 cm depth after the afforestation of farmland.

**Figure 7:**
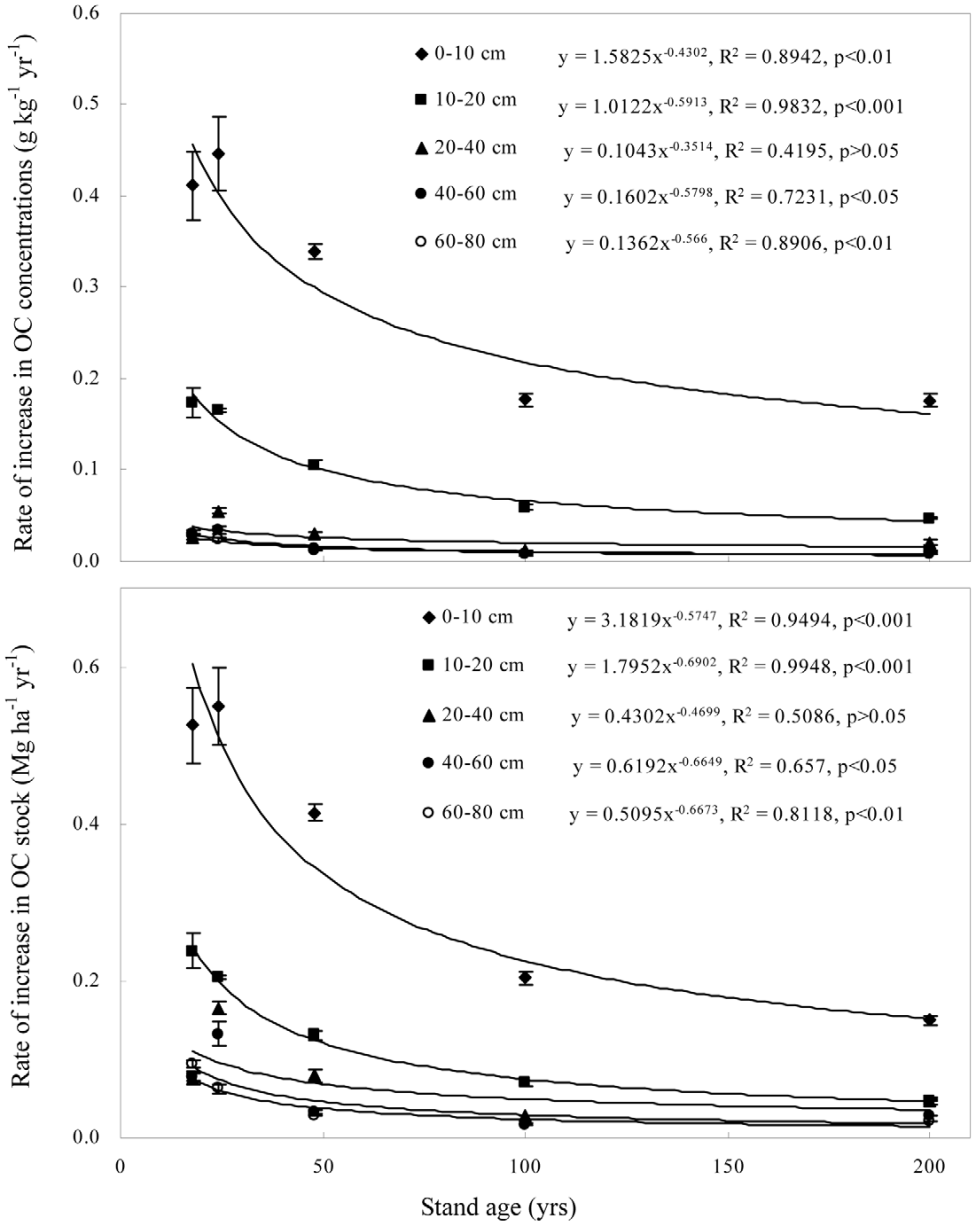
Effect of stand age on the rate of increase in forest-derived OC (the error bars are standard deviation of the means).

The rate of increase in forest-derived OC concentrations decreased nonlinearly with stand age (Fig. 7). For example, the rate of increase in forest-derived OC concentrations in the 0–10 cm depth slowed from 0.41 g kg^−1^ yr^−1^ in 18 yr stands to 0.18 g kg^−1^ yr^−1^ in 100 yr stands and 0.17 g kg^−1^ yr^−1^ in 200 yr stands. For the 10–20 cm depth, the rate of increase slowed from 0.18 g kg^−1^ yr^−1^ in 18 yr stands to 0.06 g kg^−1^ yr^−1^ in 100 yr stands and 0.05 g kg^−1^ yr^−1^ in 200 yr stands. For the 40–60 cm depth, the rate of increase slowed from 0.031 g kg^−1^ in 18 yr stands to 0.012 g kg^−1^ in 100 yr stands and 0.011 g kg^−1^ in 200 yr stands. For the 60–80 cm depth, the rate of increase slowed from 0.029 g kg^−1^ yr^−1^ in 18 yr stands to 0.012 g kg^−1^ yr^−1^ in 100 yr stands and 0.008 g kg^−1^ yr^−1^ in 200 yr stands. The accumulation rate of forest-derived OC stocks followed a similar pattern with that of forest-derived OC concentrations. The changes described above suggest that the accumulation rate of forest-derived OC will reach a constant value for each depth as the forests age.

### Slight decrease in original, crop-derived OC

**Figure 8:**
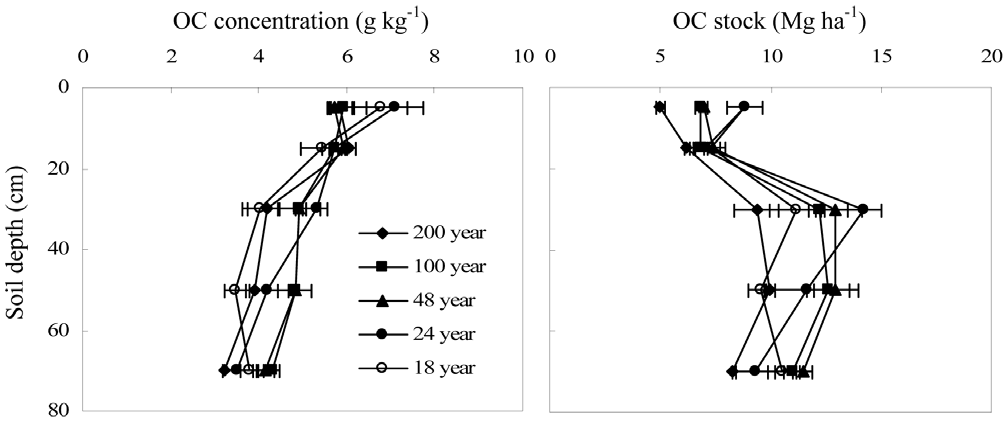
Vertical distribution of crop-derived OC (C_4_-C) concentrations and OC stocks in afforested farmland (the error bars are standard deviation of the means).

**Figure 9:**
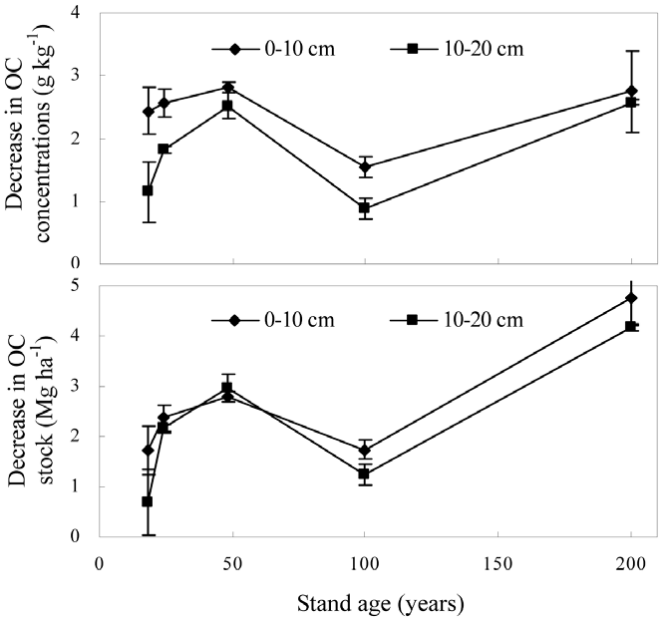
The decrease in OC concentration and OC stock in soils after afforestation of farmland (the error bars are standard deviation of the means).

The concentrations of original crop-derived OC (C_4_-C) in the topsoil of 200 yr stands were not significantly different than those of other stand ages. However, crop-derived OC stocks were lower in the 200 yr stands than those of other stand ages because the soil bulk density in the 200 yr stands was less than those of the other stand ages (Fig. 8). There was no significant difference in original OC for different stand ages in the 0–80 cm depth and small decrease of original OC in 0–20 cm depth (Fig. 9), indicating a slight decline in original OC after the afforestation of farmland. Based on our previous assumption that OC in current farmland is equal to that when the forests were established, we calculated that the original OC concentrations in afforested soils decreased across the five forest ages at the rate of 0.064 g kg^−1^ yr^−1^ in the 0–10 cm depth and 0.044 g kg^−1^ yr^−1^ in the 10–20 cm depth. The original OC stocks decreased at the rate of 0.057 Mg ha^−1^ yr^−1^ in the 0–10 cm depth and 0.048 Mg ha^−1^ yr^−1^ in the 10–20 cm depth. The original OC in the subsoil (20–80 cm depth) of the afforested sites was slightly greater than that of current farmland (with slopes less than 1 in Fig. 6). This suggests that afforestation protects the original OC in the subsoil more than modern agricultural practices. The MRTs for original farmland C after afforestation were 330 yrs in the 0–10 cm depth, 342 yrs in the 10–20 cm depth, and 476 yrs in the 0–80 cm depth. The corresponding T_1/2_ values were 229 yrs in the 0–10 cm depth, 237 yrs in the 10–20 cm depth, and 330 yrs in the 0–80 cm depths. These values indicate a slow decrease of original farmland OC and suggest that the original farmland C is relatively stable in forests.

## Discussion

Afforestation of cultivated soil increased total soil OC concentrations in the 0–40 cm depth by 88% and soil OC stocks by 81% when averaged across all stand ages. In comparison, other studies report that afforestation increased soil OC in the 0–40 cm depth by 26–53% [5], [11], [18]. The differences between our study and others might be due to differences in stand age. When the data from the 18, 24, and 48 yr stands were averaged, we observed a 52% increase in soil OC concentration and a 60% increase in OC stock. Soil OC concentrations in the 100 and 200 yr stands averaged 143% higher than that in farmland soil. Soil OC stocks averaged 112% higher in the 100 and 200 yr stands than in farmland soil. This demonstrates the large potential for the accumulation of OC in mineral soil due to afforestation.

There was a nonlinear increase in total soil OC with stand age. The rate of increase slowed across time and eventually leveled out 100 yrs after afforestation, consistent with findings that soils continuously accumulate carbon in old-growth stands that have non-steady carbon balances [30], [31]. This result is also consistent with the statement by Luyssaert *et al* (2008) that old-growth forests can act as global carbon sinks [32]. The rates of accumulation in our study decreased with soil depth and were lower than those observed by Zhou *et al* (2006) (0.61 Mg ha^−1^ yr^−1^) for the 0–20 cm depth at a warmer site (i.e., Dinghushan, China) but higher than those by Tang *et al* (2009) (0.04 Mg ha^−1^ yr^−1^) for the 0–60 cm depth at a colder site (i.e., northern Wisconsin and the upper peninsula of Michigan) [30], [31]. The large differences in the stable accumulation rate may be due to different climatic conditions at the study sites [8], [11] as well as the differences in the total soil OC concentration at the time of afforestation. The OC accumulation rate and the fate of original carbon will probably depend on the initial OC concentrations and OC stocks. Soil OC concentrations were low at the time of afforestation in our study. In this case, we might expect rapid initial increases in soil OC concentrations after afforestation.

Our results indicated that total soil OC stocks were still increasing 100 and 200 yr after afforestation, although the rate of increase slowed across time. This observation is different from the report that soil OC stocks increased steadily at a rate of 0.13 Mg ha^−1^ yr^−1^ in the 0–30 cm depth of a California soil for 82 yr after afforestation and then leveled out [33]. Furthermore, the net increase in the total soil OC of the California soil was less than the net increase in our study (10 Mg ha^−1^ vs 23.8 Mg ha^−1^). One explanation is that climate conditions or soil properties limited the ability of the California soil to sequester C.

Many researchers have studied the changes in soil OC after afforestation of farmland [5], [11], [18], but less attention has been given to the dynamics of the original OC in afforested soils. Such research is essential for understanding how soil OC responds to the afforestation of abandoned farmland. We used the natural ^13^C abundance of soil organic matter to distinguish between crop- and forest-derived C in the afforested soils. The natural abundance ^13^C technique has been used in long-term studies about soil OC dynamics [34]–[36]. An important assumption of this technique is that the isotopic composition of SOM closely resembles the isotopic composition of the vegetation from which it was derived [37], [38]. Isotope fractionation during primary plant production or organic matter decomposition [39], [40] could affect the interpretation of our results. For example, if the δ^13^ value of the soil OC increased during mineralization, then our calculations of forest-derived OC concentrations and stocks would be too low and our calculations of crop-derived OC would be too high. Our results could also be affected by the observation that the δ^13^ value of organic matter increases (i.e. becomes more positive) with soil depth relative to the forest litter layer [41]. In our study, a 1‰ increase in the δ^13^ value of OC at the 60–80 cm depth would underestimate forest-derived OC concentrations by 11% in the 18 yr stand and 12% in the 200 yr stand.

For the 0–20 cm depth, we observed a 17% decrease in crop-derived OC concentrations 100 yr after afforestation and a 31% decrease in crop-derived OC after 200 yrs of afforestation. For the 20–80 cm depth, crop-derived OC concentrations did not change after afforestation. The changes in original farmland OC after afforestation in our study were relatively small compared with changes in original C after conversion from forest to farmland or grassland. For example, Zach *et al* (2006) reported a 33–57% decrease in original soil OC concentrations after 12–18 yrs of continuous cultivation in central Argentina [24]. Osher *et al* (2003) observed significant decreases in the original OC concentration when a natural forest in Hawaii was converted to pasture or sugarcane cultivation [22]. Powers & Veldkamp (2005) reported that the original OC concentration of the 0–10 cm depth decreased when forest was converted to pasture in northeastern Costa Rica [23]. Similarly, the MRT and T_1/2_ of original crop-derived OC in our study were also larger than those of original OC in other studies [28]. One explanation for the relatively small changes in crop-derived OC after afforestation in our study is that the OC was physically protected by soil structure that developed after afforestation. Alternatively, soil OC concentrations in the farmland were low. The crop-derived OC may have been protected through sorption on to mineral particles.

Our results show that forest-derived OC increased nonlinearly in afforested farmland. Furthermore, the accumulation rate of forest-derived OC in the soil decreased as stand age increased. These results indicate a stand age after which the forest-derived C increases at a low but stable rate. Additionally, our observations of large increases in forest-derived OC and slow losses of original crop-derived OC in afforested farmland suggest that the accumulation of OC in mineral soils is mainly influenced by the input of forest-derived OC rather than the loss of original OC, especially in the subsoil.
